# Modelling the effects of glucagon during glucose tolerance testing

**DOI:** 10.1186/s12976-019-0115-3

**Published:** 2019-12-12

**Authors:** Ross A. Kelly, Molly J. Fitches, Steven D. Webb, S. R. Pop, Stewart J. Chidlow

**Affiliations:** 1 Department of Applied Mathematics, Liverpool John Moores University, James Parsons Building, Byrom Street, Liverpool, L3 3AF UK; 2 School of Life Science, University of Dundee, Dundee, UK; 3 Department of Computer Science, University of Chester, Chester, UK

**Keywords:** Glucagon sensitivity, Glucagon effectiveness, Intravenous glucose tolerance test, Non-linear glucagon minimal model, Linear glucagon minimal model, Glucose-insulin-glucagon dynamics, Minimal model

## Abstract

**Background:**

Glucose tolerance testing is a tool used to estimate glucose effectiveness and insulin sensitivity in diabetic patients. The importance of such tests has prompted the development and utilisation of mathematical models that describe glucose kinetics as a function of insulin activity. The hormone glucagon, also plays a fundamental role in systemic plasma glucose regulation and is secreted reciprocally to insulin, stimulating catabolic glucose utilisation. However, regulation of glucagon secretion by *α*-cells is impaired in type-1 and type-2 diabetes through pancreatic islet dysfunction. Despite this, inclusion of glucagon activity when modelling the glucose kinetics during glucose tolerance testing is often overlooked. This study presents two mathematical models of a glucose tolerance test that incorporate glucose-insulin-glucagon dynamics. The first model describes a non-linear relationship between glucagon and glucose, whereas the second model assumes a linear relationship.

**Results:**

Both models are validated against insulin-modified and glucose infusion intravenous glucose tolerance test (IVGTT) data, as well as insulin infusion data, and are capable of estimating patient glucose effectiveness (*s*_*G*_) and insulin sensitivity (*s*_*I*_). Inclusion of glucagon dynamics proves to provide a more detailed representation of the metabolic portrait, enabling estimation of two new diagnostic parameters: glucagon effectiveness (*s*_*E*_) and glucagon sensitivity (*δ*).

**Conclusions:**

The models are used to investigate how different degrees of pax‘tient glucagon sensitivity and effectiveness affect the concentration of blood glucose and plasma glucagon during IVGTT and insulin infusion tests, providing a platform from which the role of glucagon dynamics during a glucose tolerance test may be investigated and predicted.

## Background

Glucose is the fundamental source of cellular energy, maintained in a precise range in the blood (70 - 110 mg/dl, 4-7 mM) to facilitate general body function [1, 2]. Systemic glucose concentration is tightly regulated by the pancreatic islets, which secrete several hormones that directly influence the metabolic pathways responsible for its utilisation and production [3]. Insulin and glucagon are the two most prominent hormones responsible for normoglycaemia, secreted by *β*-cells and *α*-cells respectively, in response to deviations in plasma glucose levels [4]. Insufficient secretion or hypoactivity of insulin can lead to diabetes mellitus; a metabolic disorder characterised by persistent hyperglycaemia. While diabetes has long been linked to impaired insulin secretion, recently, glucagon has received much attention with respect to its role in diabetes. Evidence suggests that hypersecretion of glucagon can dysregulate glucose homeostasis by initiating and maintaining hyperglycaemic conditions [5]. Unger and Cherrington have subsequently suggested that “glucagon excess rather than insulin deficiency, is the *sine qua non* of diabetes” [6]. While the mechanisms of glucagon regulation by glucose are still debated [7], pharmacological manipulation of glucagon release could potentially improve diabetic glucose regulation [3]. According to the world health organisation (WHO), high blood glucose will contribute to almost half of all deaths before the age of 70, with diabetes projected to be the seventh leading cause of death by 2030 [8]. Such trends undoubtedly imply an increase in strain on health services to meet patient demands [9] and as such, any methods that facilitate mechanistic understanding or aid earlier detection of people at risk of diabetes will significantly decrease the financial and healthcare burden.

The glucose tolerance test (GTT) is a common diagnostic tool used to assess pre-diabetic and diabetic conditions, by measuring changes in blood glucose and insulin after exposure to a large bolus of glucose. Such tests are available in different forms, for example, the intravenous glucose tolerance test (IVGTT) is used to estimate insulin sensitivity (s _*I*_), glucose effectiveness (s _*G*_), insulin secretion and beta cell function in diabetic patients [10]. Mathematical IVGTT models widely accompany the analysis of IVGTT results and are used to improve the understanding of blood glucose regulation, offering insights into the relationships between key components and to speculate the effects of population considerations [11].

Bolie et al. (1961) was the first to develop a mathematical model of the IVGTT, proposing a coupled system of two linear, ordinary differential equations (ODEs) that describe the behaviour of glucose and insulin in response to administered glucose [12]. This model is simple and may be solved analytically, but assumes glucose disappearance is a linear function of plasma insulin concentration and that both secretion and disappearance rates are proportional to blood glucose and insulin levels. These assumptions are highly idealised and are not sufficient to fully describe the complicated relationship that exists in glucose-insulin dynamics. Ackerman et al. (1965) also made an impact on early studies of glucose modelling by proposing a simple linear model to describe the interaction between insulin and glucose [13].

More sophisticated models were introduced in later studies, including the well-known Minimal Model, which was derived to analyse the behaviour of blood glucose during an IVGTT [14]. This model concentrates solely on glucose-insulin dynamics but considers separately the concentration of insulin in plasma and the amount of insulin dependent glucose uptake in tissue (termed interstitial). While this model is simple and cannot be solved analytically, its ability to return estimates for glucose effectiveness and insulin sensitivity, which are key parameters for diabetes diagnosis, is advantageous. Indeed, this model has been praised for its contribution to diabetology [15] and has been widely used since its inception [16].

Modern iterations of the minimal model have been adapted to better represent free fatty acid kinetics, as well as glucose dynamics, during insulin-modified intravenous glucose tolerance testing (IMIVGTT) [17]. Indeed, Thomaseth et al. evaluate how well mathematical models of glucose and free fatty acid kinetics perform in the presence of a counterregulatory response (CRR). Such a response is triggered during an IMIVGTT as a result of administration of insulin, which can induce hypoglycaemia in healthy insulin-sensitive patients. This results in the accuracy of such mathematical models that do not account for a CRR to be undermined [17]. Thomaseth et al. modified the minimal model to improve its predictions for both glucose dynamics and free fatty acid kinetics, by introducing a glucose concentration threshold as a signal for a CRR. Indeed, their results suggest that glucagon fits well as a CRR hormone within their modelling framework, while also reporting that inclusion of other CRR hormones (epinephrine, norepinephrine, growth hormone and cortisol) did not improve model predictions.

Despite the simplicity and widespread use of the Minimal Model, it does have some significant limitations. A major criticism of the model is that it delivers mathematically unrealistic results [18], predicting that interstitial insulin activity becomes infinite over long time-periods [19]. These authors subsequently developed a non-linear model of the IVGTT which again, considers glucose-insulin dynamics only, but possesses a steady state solution that all model solutions converge. Another drawback of the Minimal Model is that it does not consider the effects of glucagon, preventing it from completely representing the full metabolic portrait of an individual. However, this is understandable as the role of glucagon with respect to diabetes became prevalent long after the inception of the minimal model. Comprehensive models of glucose metabolism that include regulation via insulin, glucagon and epinephrine do exist [20, 21], however, such models are considerably more complex and are often deployed to probe bioenergetic mechanisms, rather than glucose dynamics during glucose tolerance testing. The role of glucagon becomes crucial when blood glucose levels are low as it ensures that a sufficient amount of glucose is produced in order to avoid unconsciousness, brain damage and the other risks posed by hypoglycaemia. The risk of hypoglycaemia is increased for diabetics, due to either an impaired response of the alpha cells in the pancreas [22], or as a side effect of insulin therapy [23] and can require an additional supply of exogenous glucagon to be administered.

This study aims to investigate the interaction between glucose, insulin and glucagon during a clinical test by developing two new mathematical models that focus exclusively on glucose-insulin-glucagon dynamics. Both models are designed to simulate the perturbations in the blood-glucose regulatory system, caused by a rapid infusion/injection of either glucose, insulin or glucagon. As a result, both models are able to accurately represent behaviour during an IVGTT and during tests that involve the intravenous infusion of insulin. Consequently, IVGTT and insulin-infusion data is used to validate the accuracy of both models.

Two new parameters, termed glucagon effectiveness and glucagon sensitivity, are defined in this paper and both quantities help to determine a patient’s responsiveness to glucagon. This work investigates the response of normal and diabetic patients to exogenous infusions of insulin, to determine how inter-individual variation in glucagon sensitivity/effectiveness potentially affects a patient’s ability to re-stabilise their blood glucose concentration to a safe level.

## Methods

**Fig. 1:**
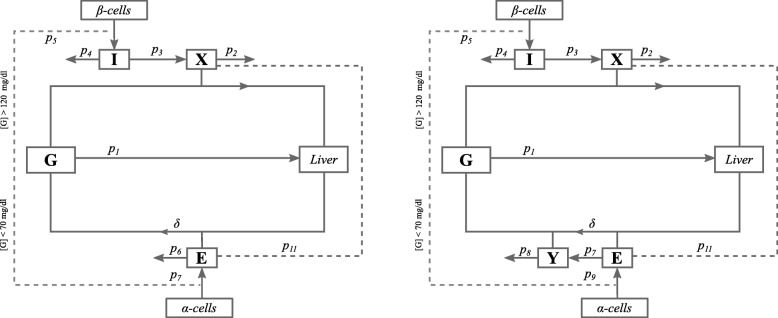
Model schematics. Model schematics for the linear glucagon minimal model (LGMM) and non-linear minimal model (NLGMM). Model variables are described as: G, glucose; I, plasma insulin; X, active insulin; E, plasma glucagon and Y, active glucagon. Solid lines depict processes whereas dashed lines depict regulatory-dependent events. Parameter values are described in Table 1. Both models describe the hormonal regulation of plasma glucose concentration during hyperglycaemia ([G] > 120 mg/dl) and hypoglycaemia ([G] < 70 mg/dl), with the NLGMM additionally considering interstitial glucagon dynamics, [E], [Y], whereas the LGMM assumes a linear relationship whereby plasma glucose will increase in proportion to the concentration of glucagon, [E], in the plasma above the basal level

The models presented in this work describe the interactions between glucose and insulin in the same way as the Minimal Model [16], but incorporate additional equations to describe glucose-insulin-glucagon dynamics (Fig. 1). The two models, however, treat the interactions between glucagon and glucose very differently.

**Table 1:** A description of the different variables and parameters that appear within both the LGMM and NLGMM

Symbol	Description	Unit
*G*(*t*)	Plasma Glucose concentration at time *t*	mg /dl
*I*(*t*)	Plasma Insulin concentration at time *t*	*μU*/ml
*X*(*t*)	Interstitial Insulin activity at time *t*	min ^−1^
*Y*(*t*)	Interstitial Glucagon activity at time *t*	min ^−1^
*E*(*t*)	Plasma Glucagon concentration at time *t*	pg /ml
*G*_*b*_	Baseline plasma glucose concentration	mg /dl
*I*_*b*_	Baseline plasma insulin concentration	*μU*/ml
*E*_*b*_	Baseline plasma glucagon concentration	ng /l
*G*_0_	Theoretical value of plasma glucose concentration at time *t*=0	mg /dl
*I*_0_	Theoretical value of plasma insulin concentration at time *t*=0	*μU*/ml
*p*_1_	Glucose Effectiveness	min ^−1^
*p*_2_	Rate of clearance of interstitial insulin	min ^−1^
*p*_3_	Rate of excess plasma insulin stimulated glucose activity	min ^−2^(*μU*/ml) ^−1^
*p*_4_	Rate of insulin disappearance from plasma	min ^−1^
*p*_5_	Rate of second phase insulin secretion (glucose dependent)	*μU*/ml min ^−2^(mg /dl) ^−1^
*p*_6_	Rate of glucagon disappearance from plasma	min ^−1^
*p*_7_	Rate of excess plasma glucagon stimulated glucagon activity	ng /l min ^−2^(mg /dl) ^−1^
*p*_8_	Rate of clearance of interstitial glucagon	min ^−1^
*p*_9_	Rate of excess plasma glucagon stimulated glucose activity	min ^−1^(ng /l) ^−1^
*p*_11_	Maximum rate at which insulin suppresses glucagon secretion	ng /l min ^−1^
*δ*	Glucagon effectiveness	mg /dl min ^−1^ (ng /l) ^−1^

### Non-linear glucagon minimal model formulation (NLGMM)

The first system extends the Minimal Model and assumes a complex, non-linear relationship between glucose and glucagon, and glucagon and insulin. This model is therefore denoted as the Non-Linear Glucagon Minimal Model (NLGMM).

The NLGMM consists of the following equations:

1 $$\begin{array}{*{20}l} \frac{d G}{dt}&=-p_{1}(G-G_{b})+(Y-X)G+G_{\text{inf}}(t), \end{array} $$

2 $$\begin{array}{*{20}l}  \frac{d X}{dt}&=-p_{2}X+p_{3}(I-I_{b}), \end{array} $$

3 $$\begin{array}{*{20}l} \frac{d I}{dt}&=-p_{4}(I-I_{b})+p_{5}(G-G_{b})^{+}t+I_{\text{inf}}(t), \end{array} $$

4 $$\begin{array}{*{20}l} \frac{d E}{dt}&=-p_{6}(E\,-\,E_{b})\,+\,p_{7}(G_{b}\,-\,G)^{+}t\,-\,p_{11}\tanh\left(\alpha(I-I_{b})\right)\!, \end{array} $$

5 $$\begin{array}{*{20}l} \frac{d Y}{dt}&=-p_{8}Y+p_{9}(E-E_{b})^{+}. \end{array} $$

When modelling an IVGTT, the NLGMM is subject to the following initial conditions

6 $$\begin{array}{*{20}l} G(0)&=G_{0}, \qquad X(0)=0, \\ &\qquad I(0)=I_{0}, \qquad E(0)= E_{b}, \qquad Y(0)=0. \end{array} $$

All parameters are positive and variables appearing within the model are defined in Table 1. Note that the positive superscript used in the system above is shorthand notation for the following function

7 $$\begin{array}{*{20}l} (G-G_{b})^{+}= \left\{\begin{array}{ccc} G-G_{b}, \qquad G\geq G_{b},\\ 0, \qquad G<G_{b}. \end{array}\right. \end{array} $$

A similar definition is used for the functions (*G*_*b*_−*G*)^+^ and (*E*−*E*_*b*_)^+^. In addition, the functions *G*_inf_(*t*) and *I*_inf_(*t*) are used to account for external infusions of glucose and insulin into the body and can change dramatically in different tests.

The NLGMM accounts for the concentration of glucagon in plasma but also accounts for the effects of glucagon in tissue, termed active glucagon. The idea behind this model is that the amount of plasma glucagon is irrelevant. Instead, it is the amount able to stimulate endogeneous glucose production that directly raises the concentration of glucose in the blood stream. This assumption is useful as it allows patients who suffer from hyperglucagonemia to be easily accounted for and was first suggested by [22] as a suitable mechanism for glucose-glucagon dynamics.

The concentration of active glucagon is dependent upon plasma glucagon and will only increase if the concentration of plasma glucagon is above its basal value. If this criterion is met, there will be more active glucagon present in the system and endogeneous glucose production will increase. However, if the concentration of blood glucose becomes too high, the concentration of active glucagon will decrease to zero due to the lack of secretion of plasma glucagon and thus endogeneous glucose production will cease.

In terms of modelling the concentration of glucagon in plasma, this model assumes that glucagon is only released from the pancreas when glucose concentration falls below its pre-test, basal level. It further assumes that high levels of insulin in plasma suppress glucagon secretion and cause the concentration in plasma to fall. This phenomenon has been observed in the work of [24] and should be accounted for in any mathematical representation of this system. The term accounting for this interaction between hormones does not allow the rate of change of glucagon to continually decrease in the presence of increasing insulin but rather, it assumes that beyond a certain insulin concentration, glucagon secretion will decrease at a constant rate.

### Linear glucagon minimal model formulation (LGMM)

The second model presented assumes that the concentration of glucose is directly affected by plasma glucagon and therefore omits interstitial glucagon activity. This system is referred to as the Linearised Glucagon Minimal Model (LGMM), as the rate of change of glucose depends in a linear fashion on the concentration of plasma glucagon.

The system of equations for the LGMM is

8 $$\begin{array}{*{20}l} \frac{d G}{dt}&=-p_{1}(G-G_{b})-XG+\delta(E-E_{b})+G_{\text{inf}}(t), \end{array} $$

9 $$\begin{array}{*{20}l} \frac{d X}{dt}&=-p_{2}X+p_{3}(I-I_{b}),\\  \frac{d I}{dt}&=-p_{4}(I-I_{b})+p_{5}(G-G_{b})^{+}t+I_{\text{inf}}(t), \end{array} $$

10 $$\begin{array}{*{20}l} \frac{dE}{dt}&\!\,=\,-p_{6}(E\,-\,E_{b})\,+\,p_{7}(G_{b}\,-\,G)^{+}t\,-\,p_{11}\!\tanh\big(\alpha(I\,-\,I_{b})\big). \end{array} $$

In the case of an IVGTT, the LGMM is solved subject to the corresponding initial conditions

11 $$\begin{array}{*{20}l} G(0)&=G_{0}, \qquad X(0)=0,\\ & \qquad I(0)=I_{0}, \qquad E(0)=E_{b}. \end{array} $$

The new parameter *δ* is defined in Table 1.

In the LGMM, the concentration of plasma glucagon is modelled in the same way as in the NLGMM, but the rate of change of glucose concentration is instead assumed to be directly proportional to the concentration of plasma glucagon. In this model, a fall in plasma glucagon concentration will immediately lead to a rise in the concentration of blood glucose, whereas an increase in plasma glucagon will lead to an immediate rise in glucose concentration.

### Physiological parameters

One of the principal advantages of retaining the glucose-insulin dynamics as described by the Minimal Model is that the glucose effectiveness (*s*_*G*_) and insulin sensitivity (*s*_*I*_) of a patient may be estimated. Hence, estimates of these parameters are recovered from the following equations:

12 $$\begin{array}{*{20}l} s_{G} &= p_{1}, \end{array} $$

13 $$\begin{array}{*{20}l} s_{I} &=\frac{p_{3}}{p_{2}}. \end{array} $$

The reader is referred to [14] for more information about how these estimates are derived. All three of these parameters (*p*_1_−3) are common to the NLGMM and LGMM, allowing both models to compute approximations to these key parameters.

As the interactions between glucagon and glucose are modelled in a different way, both models return different estimators of the glucagon effect. Following a similar idea to that used in [16] to compute insulin sensitivity, if a non-zero steady state value of glucagon activity is achieved, it then follows from Eqs. (5) and (1) that:

14 $$\begin{array}{*{20}l} Y = \frac{p_{9}(E-E_{b})}{p_{8}} \end{array} $$

and

15 $$\begin{array}{*{20}l} \frac{d G_{SS}}{d t} = -p_{1}(G-G_{b})-XG+\frac{p_{9}(E-E_{b})}{p_{8}}G, \end{array} $$

where the subscript denotes “steady state”, and corresponds to the rate of change of glucose when the concentration of active glucagon is steady. The glucagon Sensitivity (*s*_*E*_) of a patient may then be defined as:

16 $$\begin{array}{*{20}l} s_{E}=\frac{\partial^{2}}{\partial G\partial E}\left(\frac{d G_{SS}}{dt}\right)=\frac{p_{9}}{p_{8}}. \end{array} $$

This is identical to the result given in [22].

The LGMM does not contain the variable representing active glucagon and is therefore unable to return an estimate of glucagon sensitivity. However, it is possible to derive an alternate parameter that allows the effects of glucagon to be quantified.

Using (8), let us define the function

$$\begin{array}{*{20}l} F(G,X,E) = -p_{1}(G-G_{b})-XG+\delta(E-E_{b})  \end{array} $$

which describes the rate at which the concentration of plasma glucose changes. Taking the derivative of this function with respect to *G* gives

17 $$\begin{array}{*{20}l} \frac{\partial F}{\partial G}=-p_{1}=-s_{G}. \end{array} $$

This quantity describes the rate at which the concentration changes according to the amount of glucose present in the system and is equivalent to the glucose effectiveness. According to [14], ‘glucose effectiveness is defined as the enhancement of glucose disappearance due to an increase in the plasma glucose concentration’. The appearance of the minus sign within the equation above explains why glucose effectiveness is used to describe the rate of disappearance as it cause the concentration to decrease.

Taking the partial derivative of (17) with respect to *E* yields

$$\begin{array}{*{20}l} \frac{\partial F}{\partial E}=\delta. \end{array} $$

This quantity describes the rate at which the concentration of glucose changes according to the amount of glucagon present in the system. It is therefore appropriate to refer to this quantity as glucagon effectiveness. Using the definition provided above for glucose effectiveness, the glucagon effectiveness parameter is defined as the quantitative enhancement of glucose appearance due to an increase in plasma glucagon concentration.

Clearly, although glucagon effectiveness and glucagon sensitivity are derived in different ways and defined differently, they both allow a patient’s response to glucagon to be characterised and may be used to quantify how responsive a patient is to glucagon. (discussed later in the manuscript).

### Model solutions

A detailed analysis of the qualitative behaviour of solutions of the LGMM and NLGMM may be found in the Appendix. The LGMM consists of a system of four non-linear differential equations and a maximum of 10 unknown parameters, whilst the NLGMM consists of five non-linear differential equations and a maxmimum of 11 unknown parameters. As a result of the complexity of both systems, both are solved numerically in MATLAB using the ODE45 solver. The unknown parameters are fitted to experimental data using LSQNONLIN, a non-linear least squares solver, during the solution process.

## Results and discussion

### Model validation

The accuracy of solutions produced from both the LGMM and NLGMM were validated against patient data extracted from Thomaseth *et al* [17] before being used to make new predictions. As the LGMM and NLGMM are designed to be able to model rapid infusions of glucose, insulin or glucagon, both models were validated during two different types of medical test: an IVGTT, and in a test that artificially induces hypoglycemia via intravenous infusions of insulin.

### Validation against an IVGTT

Model solutions from the LGMM and NLGMM were compared against experimental data extracted from [17], whereby an Insulin Modified IVGTT (IM-IVGTT) and a modified test (GC-IM-IVGTT) was performed on thirteen patients. Briefly, insulin-sensitivity can be probed by the administration of insulin during an IM-IVGTT, which can cause transient hypoglycemia in healthy insulin-sensitive patients. The GC-IM-IVGTT however, is a modified IM-IVGTT test, which includes a glucose infusion, or “glucose clamp (GC)”, in order to prevent hypoglycemia. The two different tests are described by [17] as follows:

“Thirteen nondiabetic volunteers [7 male and 6 female, aged between 25 and 27 years old, with a body mass index (BMI): 22.1±0.7 kg /m^2^, (*mean*±*SD*)] were studied in random order during standard IM-IVGTT: 0.3 g/kg glucose at time 0 and 0.03 IU/kg insulin at 20 min and during a modified test (GC-IM-IVGTT) with additional glucose infusion adjusted manually to prevent plasma glucose concentration from falling below 100 mg/dl. Insulin, glucose, and NEFA plasma concentrations were measured at frequent intervals, from 15 min before the beginning of the test and during the following 3 h. Plasma concentrations of C-peptide, glucagon, cortisol, growth hormone, epinephrine, and norepinephrine were also measured at timed intervals.”

The aim of the investigation by [17] was to investigate how nonesterified fatty acids affect the concentration of glucose during an IVGTT. However, the authors provide average patient concentrations of glucose, insulin and glucagon throughout the IM-IVGTT and GC-IM-IVGTT which allows a thorough comparison of predictions from both the LGMM and NLGMM to all three quantities. The rational for using the data from Thomaseth et al. was two-fold: first, the vast majority of research papers in the available literature that utilise IVGTTs in an investigation do not contain any plasma glucagon data, and second, comparing model performance against data obtained from two different types of IVGTT provides a more complete model validation. It is worth noting that while the data extracted from Thomaseth et al. was used to validate the model, the adaptations the authors made to the minimal model were not deployed in this work because the consideration of free fatty acid kinetics or counterregulatory responses are not prominent here. Furthermore, the minimal model has been ameliorated numerous times since its inception for different specific outputs. Which amendments to include therefore, are a function of the desired output.

Modelling both an IM-IVGTT and GC-IM-IVGTT is more complicated than a standard IVGTT as the additional infusions of glucose and insulin that are administered during the test must be incorporated within the mathematical models. The reader is referred to the Appendix for a full description of how this is conducted here.

**Fig. 2:**
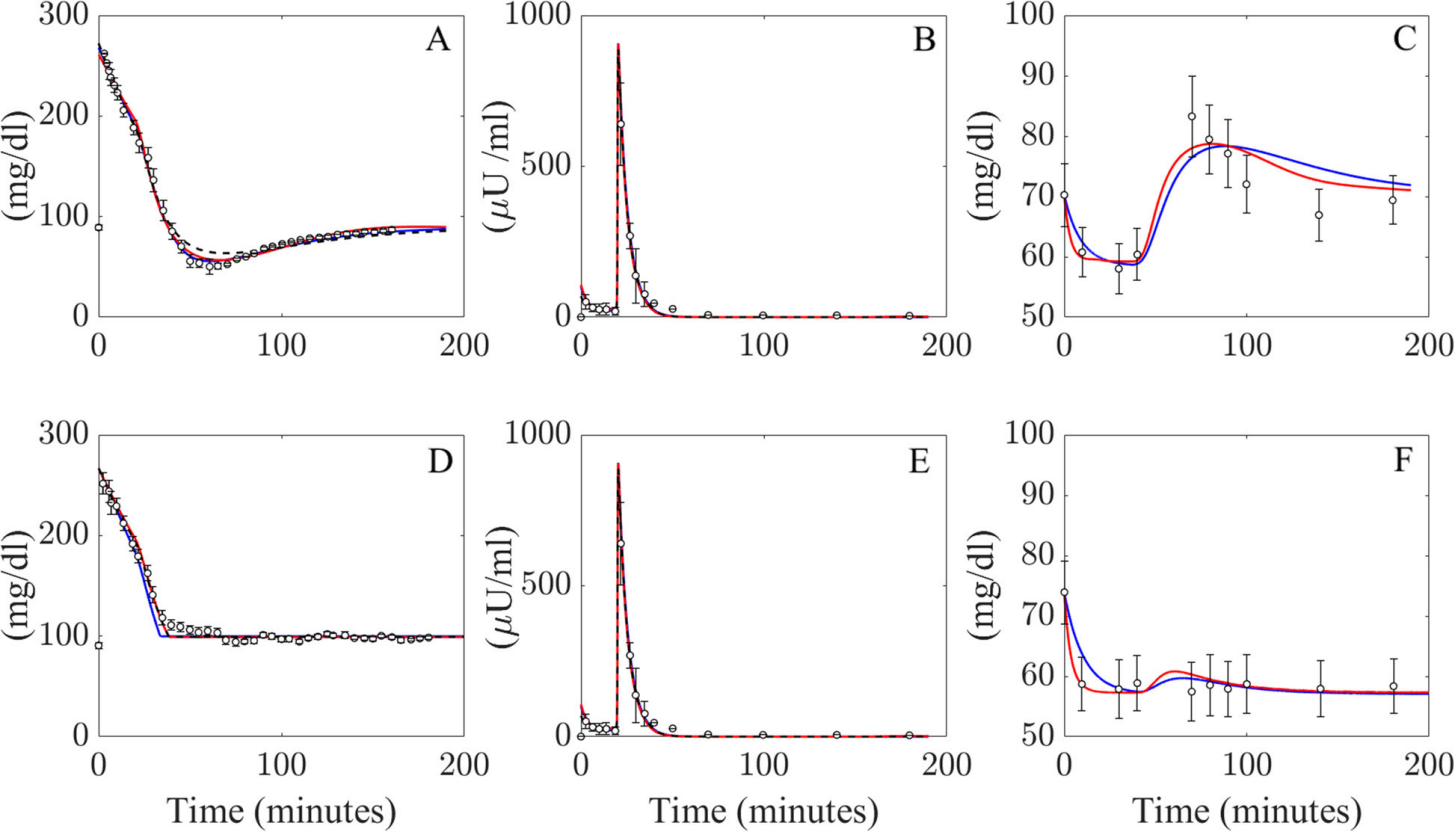
Model validation. Model simulations produced by the LGMM (blue lines), NLGMM (red lines) and the minimal model (black dashed lines) for the two different IVGTT’s (mean data and SEM illustrated by circles and error-bars) presented in Thomaseth et al. 2014 [17]. A, B and C shows the predictions for the insulin modified IVGTT, while D, E and F illustrates the results for the IVGTT with glucose infusion. A and D represent blood glucose concentration, B and E represent plasma insulin and C and F represent plasma glucagon concentration’

Figure 2 compares the patient data from [17] to model solutions for the LGMM, NLGMM and the Minimal Model. All three model simulations fit the glucose and insulin data well, while the LGMM and NLGMM provide a good representation of the glucagon data. The function representing insulin infusion replicates the actual dose well in the IM-IVGTT but overestimates the amount given in the GC-IM-IVGTT. The simulated predictions of glucagon from both models fit the data well, and pass through the majority of the errorbars indicating good accuracy. The goodness of fit values computed from all three models in this example are contained within Table 2 and indicate that all models provide highly accurate solutions here.

**Table 2:** Goodness of fitness values (*R*^2^) of all model simulations presented in Fig. 2

Dataset	Model	Glucose	Insulin	Glucagon
(IM-IVGTT)	LGMM	0.994	0.984	0.779
	NLGMM	0.997	0.993	0.850
	MinMod	0.990	0.990	-
(GC-IM-IVGTT)	LGMM	0.981	0.799	0.841
	NLGMM	0.990	0.754	0.937
	MinMod	0.992	0.766	-

### Validation against hypoglycemic data

LGMM and NLGMM simulations were also compared to the results of Bolli *et al.*, presented in [25]. The aim of the investigation by these authors was to determine the role of intraislet insulin in the response of glucagon to hypoglycemia. In the experiments conducted in this work, hypoglycemia was artificially induced in both a control group and a group of patients with diabetes by infusing patients with insulin intraveneously. Upon completion of the study, the authors were able to deduce that glucagon response to hypoglycemia induced by hyperinsulinemia is independent of intra-islet and circulating insulin.

The experiments within the above named work may be replicated using the models proposed here. However, the hyperinsulinemia triggered by the intravenously administered insulin must be modelled separately and provided as an additional input to the LGMM, NLGMM and Minimal Model.

According to [25], the participants in the study are described as follows:

“Seven normal healthy volunteers within 10% of ideal body weight and five age- and weight-matched insulin-dependent diabetic subjects were studied after obtaining fully informed consent. The normal subjects, ranging in age from 19 to 35 years (26 ± 3 years, mean ±SEM), had been on a weight-maintaining diet (300 g carbohydrate/d) for at least 1 week before all studies. The diabetic subjects had diabetes of 13-15 month duration and were C-peptide deficient (0.08 ± 0.02 ng/ml before and 0.08 ± 0.04 ng/ml after 1 mg glucagon given intravenously).”

The experimental studies that are referred to in this paper concern both the control and diabetic group being infused with insulin intravenously at a rate of 30mU /*m*^2^ per minute for an hour from the fasting state. Blood glucose concentrations, plasma insulin and plasma glucagon concentrations were measured at frequent intervals and population averages in both groups were taken to determine the mean group response across the test.

The Minimal Model is not designed to simulate this type of experiment as it does not account for the effects of glucagon. The LGMM and NLGMM however are suitable for predicting glucose and hormonal responses, therefore this data was chosen for validation to show their ability to simulate different tests with accuracy. The reader is referred to the Appendix for further details of how model results are produced in this example.

**Fig. 3:**
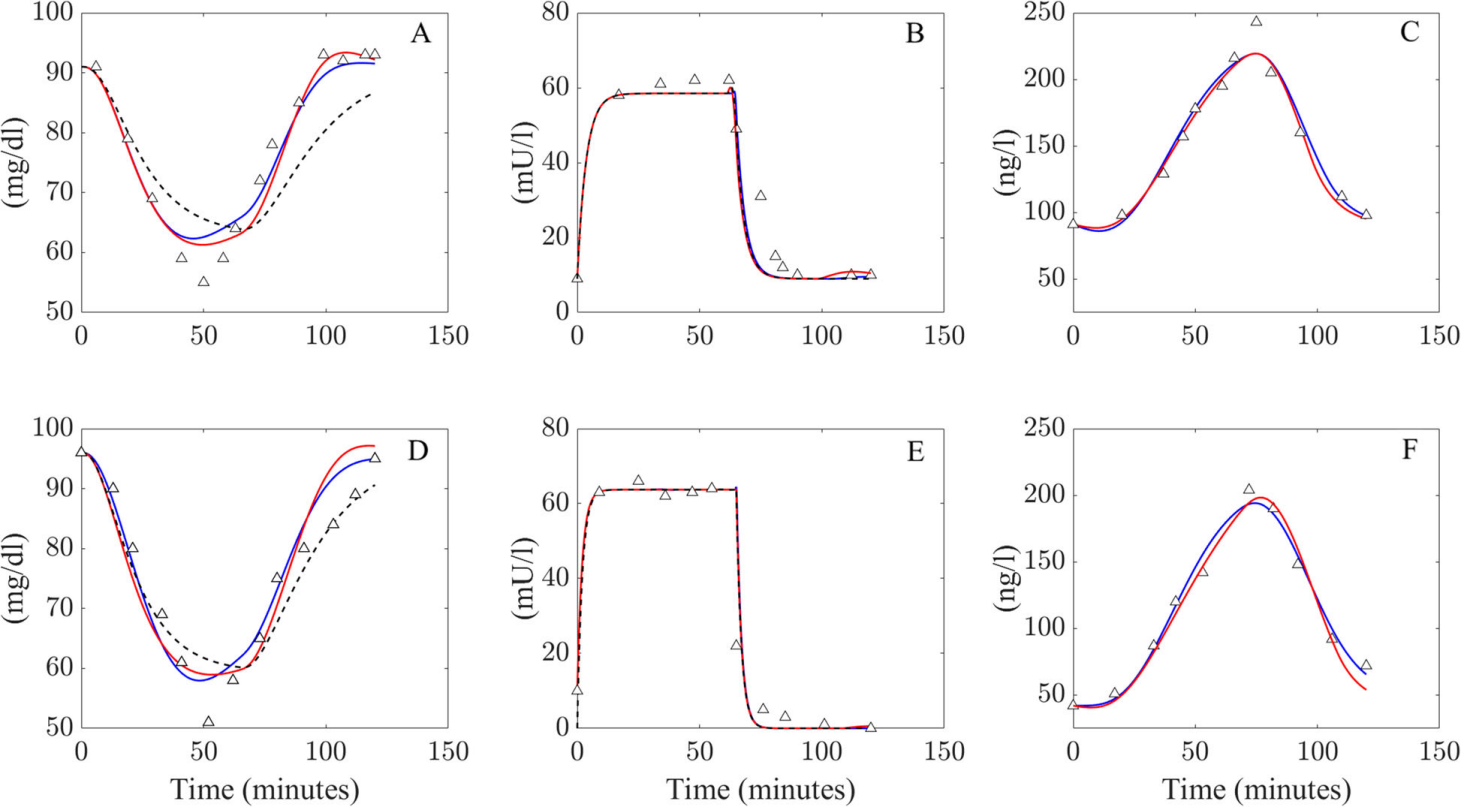
Model simulations produced from both the LGMM and NLGMM for the insulin induced hypoglycemic tests conducted by [25]. A, B and C shows the predictions for the control group, while D, E and F depicts the results for the diabetic group. Blue lines correspond to solutions from the LGMM, red lines to solutions from the NLGMM and the black dashed lines to the Minimal Model. Experimental data from [25] is shown as triangles

The original patient data as given by [25] and model solutions from the LGMM, NLGMM and Minimal Model are presented in Fig. 3, accompanied by goodness of fit values in Table 3. It is very clear that the solutions from the LGMM and NLGMM closely match the given patient data for glucose, insulin and glucagon in both the control and diabetic groups. The predicted plasma glucagon concentrations are incredibly accurate with both new models fitting the data values almost exactly. The Minimal Model struggles to fit the glucose data in the control group and performs worse than the LGMM and NLGMM. It does however provide a good fit to the glucose data in the diabetic group which is indicative of patients in this group being less sensitive to the effects of glucagon.

**Table 3:** Goodness of fitness values (*R*^2^) of all model simulations presented in Fig. 3

Group	Model	Glucose	Insulin	Glucagon
Control	LGMM	0.942	0.920	0.967
	NLGMM	0.948	0.917	0.967
	MinMod	0.666	0.921	-
Diabetic	LGMM	0.904	0.787	0.983
	NLGMM	0.846	0.792	0.969
	MinMod	0.887	0.801	-

## Model comparison and predictions

Figures 2 and 3 illustrate the ability of the LGMM and the NLGMM to provide accurate approximations during both an IVGTT and tests that induce hypoglycemia by infusing a patient with insulin. The performance of both models may now be compared in more detail to discern whether one model is significantly more appropriate than the other.

### Comparing the LGMM and NLGMM

A simple way to initially compare the performance of the two new models is by comparing values obtained from the Akaike Information Criterion (AIC) and the Bayesian Information Criterion (BIC) in the examples considered above. The AIC and BIC are penalised-likelihood criteria, often used during model selection and are representative of the distance between the fitted likelihood of a model and the unknown true likelihood function of the data. The only difference between the two measures is that the BIC penalises model complexity more heavily.

The second order AIC (AICc) can be calculated to account for smaller sample sizes which does penalise the use of additional parameters more heavily than the usual AIC. In what follows, values from all three criteria are used to compare the LGMM and NLGMM. The AIC, second order modified (AICc) and BIC values for the two validation outputs are contained in Table 4. The LGMM yields the minimum AIC values for three out of the four tests (IM-IVGTT, GC-IM-IVGTT and the insulin infusion diabetic group), with the NLGMM yielding the minimum AIC value for the insulin infusion control group. However, the AICc and BIC values corresponding to the LGMM are smaller in all cases, with significantly smaller values recorded for the IM-IVGTT and control group. The AICc considers the smaller sample size and therefore lends credence to the LGMM being the most appropriate model for the insulin infusion results. Moreover, the LGMM model possesses a smaller parameter space than the NLGMM, meaning less potential error during parameter estimation. Hence on the basis of these tests, it seems evident that the LGMM is the most appropriate model to use.

**Table 4:** Values of the AIC, modified AIC and BIC computed from the model simulations in the two examples used to validate the LGMM and NLGMM

Test type	Model	AIC	AICc	BIC
IM-IVGTT	LGMM	552.67	558.06	325.13
	NLGMM	683.07	689.57	457.64
GC-IM-IVGTT	LGMM	680.50	680.70	423.77
	NLGMM	691.62	691.82	434.89
Insulin infusion (Control group)	LGMM	277.70	286.87	168.82
	NLGMM	276.19	287.68	168.87
Insulin infusion (Diabetic group)	LGMM	262.84	273.85	170.73
	NLGMM	268.40	279.93	177.72

Another robust test that can be used to compare model performance is to determine how good both models are at accurately recreating patient profiles and model parameters. This test requires simulated data instead of real patient data so that a very large amount of tests may be run and statistically unbiased conclusions may be drawn. Using precise, known model parameters also allows the exact error in the parameter estimates to be computed.

In this example, a selection of randomly generated parameters values are input into both the LGMM and NLGMM and used to simulate blood glucose, plasma insulin and plasma glucagon profiles during an IVGTT. This data is then distorted with a specified level of noise and used to create a “virtual patient cohort" which is passed into both models. The models are then fitted to the data and used to estimate the parameters which are assumed to be unknown. The returned parameter estimates may then be directly compared to the exact values that were used originally, facilitating a comparison of model performance.

**Fig. 4:**
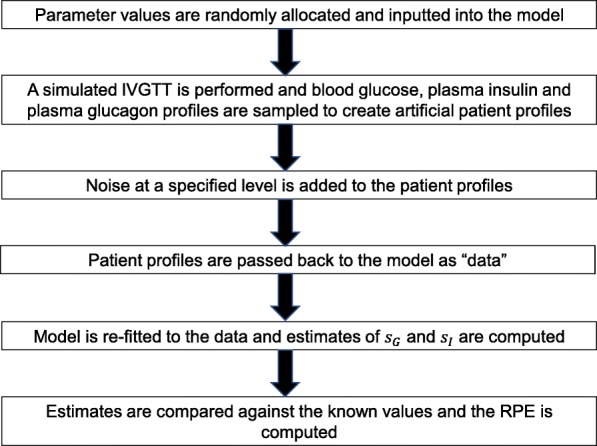
A flowchart indicating how parameter estimates are computed and compared during the comparison of the LGMM and NLGMM

As the glucose effectiveness and insulin sensitivity of a patient are of real clinical significance, this investigation focuses solely on the accuracy of the estimates obtained for these parameters. The inclusion of noise within the data represents potential errors in the way that measurements are taken, collected and/or recorded. Investigating how the estimates of glucose effectiveness and insulin sensitivity returned by both models are affected by noise will determine how viable it is to use these models when there is a reasonable degree of error in the patient data. The accuracy of the predicted values of glucose effectiveness and insulin sensitivity was investigated by considering the relative percentage error (RPE) in each approximation. The RPE in each approximation was calculated using the following formulae:

18 $$\begin{array}{*{20}l} \text{RPE}\ \text{in}\ \text{Glucose}\ \text{Effectiveness}&=\left|\frac{s_{G}-s_{G}^{A}}{s_{G}}\right|\times 100, \end{array} $$

19 $$\begin{array}{*{20}l} \text{RPE}\ \text{in}\ \text{Insulin}\ \text{Sensitivity}&=\left|\frac{s_{I}-s_{I}^{A}}{s_{I}}\right|\times 100, \end{array} $$

where the superscript *A* denotes the returned approximation to the parameter of interest. If the relative percentage error is close to zero, the returned approximation to the parameter is highly accurate. A complete description of how these simulations are conducted is contained in a flowchart within Fig. 4, representing a total of 500 simulations.

The model parameters used in this test are described fully in Table 6 in the Appendix. The chosen ranges for the parameters *p*_1_−*p*_5_ are taken from Nittala et al. [26]. However, the ranges used for the parameters *p*_6_−*p*_11_ and *δ* were chosen after empirical testing using trial and error by the authors. This consisted of using the corresponding fitted values for these parameters in example 1 as the median value of these quantities and picking a suitable range of values either side of the median that provided realistic glucose, plasma insulin and plasma glucagon behaviour.

**Fig. 5:**
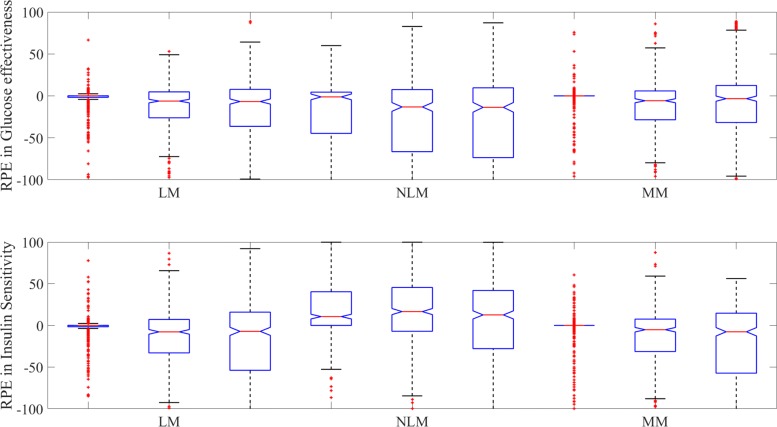
Boxplots of the estimates of glucose effectiveness and insulin sensitivity returned from the LGMM (abbreviated to LM here), NLGMM (abbreviated to NLM) and the Minimal Model (MM). The top panel shows the boxplots for the RPE in glucose effectiveness and the bottom panel shows the boxplots for the RPE in insulin sensitivity. The three boxplots above each model label represent 0%, 5%, and 10% noise respectively from left to right

Figure 5 presents a series of boxplots depicting the relative percentage error between the estimated and observed values of glucose effectiveness (*s*_*G*_) and insulin activity (*s*_*I*_) obtained from both the LGMM and NLGMM. Equivalent results obtained from the Minimal Model are also presented to allow further comparison of model performance. A series of descriptive statistics that compare the median and interquartile range of the relative percentage error produced from each model are further contained in Table 5.

**Table 5:** Statistical Comparison of the relative percentage error in the approximations to glucose effectiveness and insulin sensitivity, produced from the LGMM, NLGMM and Minimal Model

		LGMM		NLGMM		MinMod		
Noise level	Parameter	Median	IQR	Median	IQR	Median	IQR	*p*-value
0%	*s*_*G*_	0	1.7362	-1.2291	49.1232	0	0.0238	0.0031
	*s*_*I*_	0	1.5644	10.5997	40.3733	0	0.0091	< 0.0001
5%	*s*_*G*_	-6.2099	30.8770	-13.2109	73.9800	-5.8340	34.3679	0.0041
	*s*_*I*_	-7.7208	40.1055	16.6060	52.5382	-5.1386	38.8835	< 0.0001
10%	*s*_*G*_	-6.7471	44.0449	-13.6528	83.1426	-3.3342	44.1309	0.0001
	*s*_*I*_	-7.1037	69.7317	12.6253	69.7497	-7.6116	71.9373	< 0.0001

The medians of each dataset and the interquartile range (IQR) are presented here and the p-values produced from the Kruskal-Wallis test at the 95% confidence level that tests if the data from each model is obtained from the same distribution

It is evident in all boxplots that the results produced from the LGMM are far more accurate than those produced from the NLGMM. The results obtained from the LGMM consistently have a much lower spread than the NLGMM, as indicated by the much smaller box size. The interquartile range and hence box size increases as the amount of noise in the patient data increases for all models, which indicates that the accuracy of the estimates of glucose effectiveness and insulin sensitivity produced by all models decreases with noise. This is unsurprising as errors in patient data will increase the difficulty in model-fitting and lead to increased uncertainty in parameter estimation. There are also significantly more outliers obtained in this case, not all of which are shown here due to the scale chosen. However, the median values of glucose effectiveness and insulin sensitivity produced from the LGMM are still much closer to zero than those obtained by the NLGMM when there is noise in the data, and therefore the LGMM still proves to be a more accurate model in these cases.

The results produced from the Minimal Model are far more accurate than the NLGMM but comparable to those produced from the LGMM. Furthermore, the median and interquartile range produced from both LGMM and NLGMM are similar at the 5% and 10% noise levels. There is evidence however that the Minimal Model produces the more accurate approximations to glucose effectiveness and insulin sensitivity with zero noise in the patient data as the interquartile range is much smaller than that computed for the LGMM.

A more definitive comparison between the LGMM, NLGMM and Minimal Model may be obtained by comparing the predictions from all models for each dataset using the Kruskal-Wallis test. The Kruskal-Wallis test checks the null hypothesis that data from all three models originate from the same distribution against the alternative hypothesis that they do not. As can be seen in Table 5, the p-values produced in all cases for this test are significant at the 5% level and consequently, the data produced from all three models does not come from the same distribution.

The performance of the LGMM and NLGMM may be compared directly using the Mann-Whitney U-test and again, statistically significant results at the 5% level are obtained for all of the simulations produced here (not shown here). Given that the medians produced for the LGMM are much smaller than those produced for the NLGMM and that the interquartile range is persistently smaller for the LGMM, the results of these tests indicate that the approximations computed from the LGMM are statistically more accurate than the NLGMM. However, the performance of the LGMM is comparable to that of the Minimal Model.

### Investigating the response of glucagon during an IVGTT

As both models have been validated against patient data and have been compared against one another to contrast model performance, analysis concludes with an investigation into how the concentration of plasma glucagon varies during glucose tolerance testing. In this example, the response of glucagon during an IVGTT was investigated. Particular attention was given to the relationship between insulin and glucagon, in an attempt to determine how glucagon may be suppressed during periods of hyperglycemia. As a result, all parameter values in this example are fixed and set equal to the fitted parameter values obtained from the first dataset in Fig. 2, (see the Appendix for details) apart from *p*_11_, which governs how sensitive glucagon suppression is for any given concentration of insulin.

**Fig. 6:**
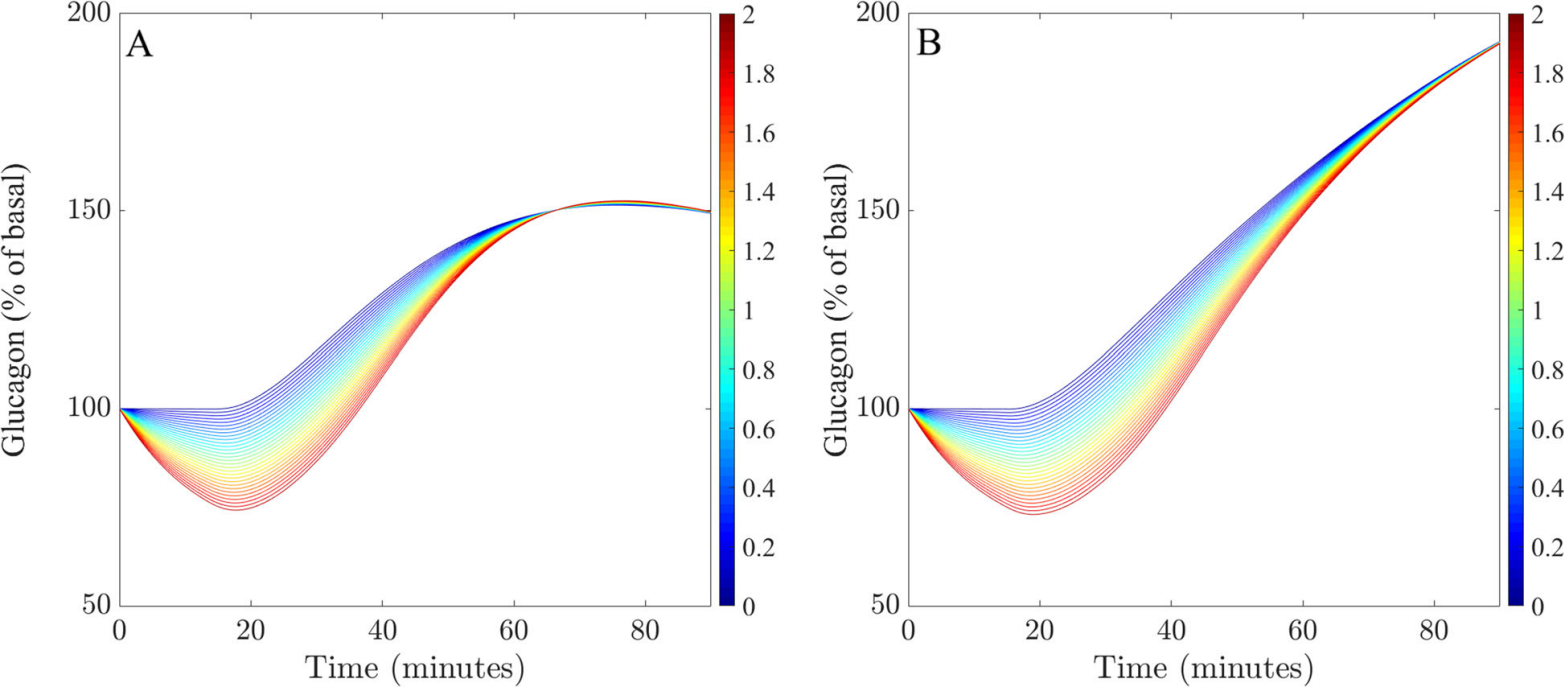
Variations in endogenous glucagon production. Endogeneous glucagon production during an IVGTT for a range of values of *p*_11_ that correspond to differing maximum rates of glucagon suppression by insulin for the LGMM (**a**) and NLGMM (**b**). Blue lines correspond to smaller values of *p*_11_ and red lines correspond to larger values. Parameter values used for the simulations are located in Table 7 in Appendix D, with the value of *p*_11_ varying between 0 and 2 in increments of 0.1 between simulations

Figure 6 illustrates how different maximum rates of insulin-dependent glucagon suppression influences glucagon concentration, during an IVGTT for the LGMM and NLGMM. In this example, blue lines correspond to smaller values of *p*_11_ which indicate relatively little glucagon suppression, and red lines corresponds to larger values, which indicate more significant glucagon suppression. Both models predict that patients with a higher sensitivity (larger *p*_11_) of insulin-mediated glucagon suppression, exhibit a lower glucagon concentration, compared to a non-sensitive patient.

Figure 6 also illustrates the fundamental differences between how the glucagon metabolism in the LGMM and NLGMM is simulated during an IVGTT. The LGMM predicts that regardless of varying degrees of insulin-mediated glucagon suppression, glucagon concentration will peak and plateau at aproximately 150% of basal, whereas the NLGMM reaches almost 200% of the basal glucagon concentration, with little sign of decreasing. Ultimately, the metabolism of glucagon hinges upon the concentration of glucose, either by direct secretion during hypoglycaemia, or indirectly via insulin-mediated inhibition during hyperglycemia. In this instance, glucagon concentration is able to recover quicker within the LGMM, due to the omission of interstitial glucagon activity, given that the rate of change of plasma glucagon is directly proportional to the concentration of glucose. The NLGMM however, does include interstitial glucagon activity, rendering the concentration of plasma glucagon a less useful metric than the amount of effective glucagon working in the system at a given time. These simulations suggest that first, manipulation of *p*_11_ within both models facilitates simulation of inter-individual variation with respect to insulin-mediated glucagon suppression, and second, that the NLGMM is perhaps better suited to simulate patients who suffer from hyperglucagonemia.

### Investigating the response of glucagon during periods of hypoglycemia

The final example presented in this work considers how glucagon response within a patient with Type 1 diabetes mellitus (T1DM) varies during periods of hypoglycemia. The most novel aspect of the two new models introduced within this work is that they both seek to describe the dynamics between glucose, insulin and glucagon, given that the relationship between glucose and glucagon is key when a patient experiences hypoglycemia.

Within this example, model simulations explored the possible variations between patients. The simulated test represents a patient with T1DM receiving an intravenous infusion of one unit of insulin in the fasting state and measures how different values of glucagon effectiveness and glucagon sensitivity affect the response of both blood glucose and plasma glucagon over a three hour period, assuming that no glucose is ingested or administered to correct sugar levels.

All parameters within this example, except for glucagon effectiveness (*δ*) in the case of the LGMM and glucagon sensitivity (*s*_*E*_) in the case of the NLGMM, are fixed and detailed within the Appendix. It should be noted that some of the parameter values used in this example may not correspond exactly to the physiological parameters that one would expect for a Type 1 diabetic so the predictions produced here should be regarded primarily as qualitative rather than quantitative.

**Fig. 7:**
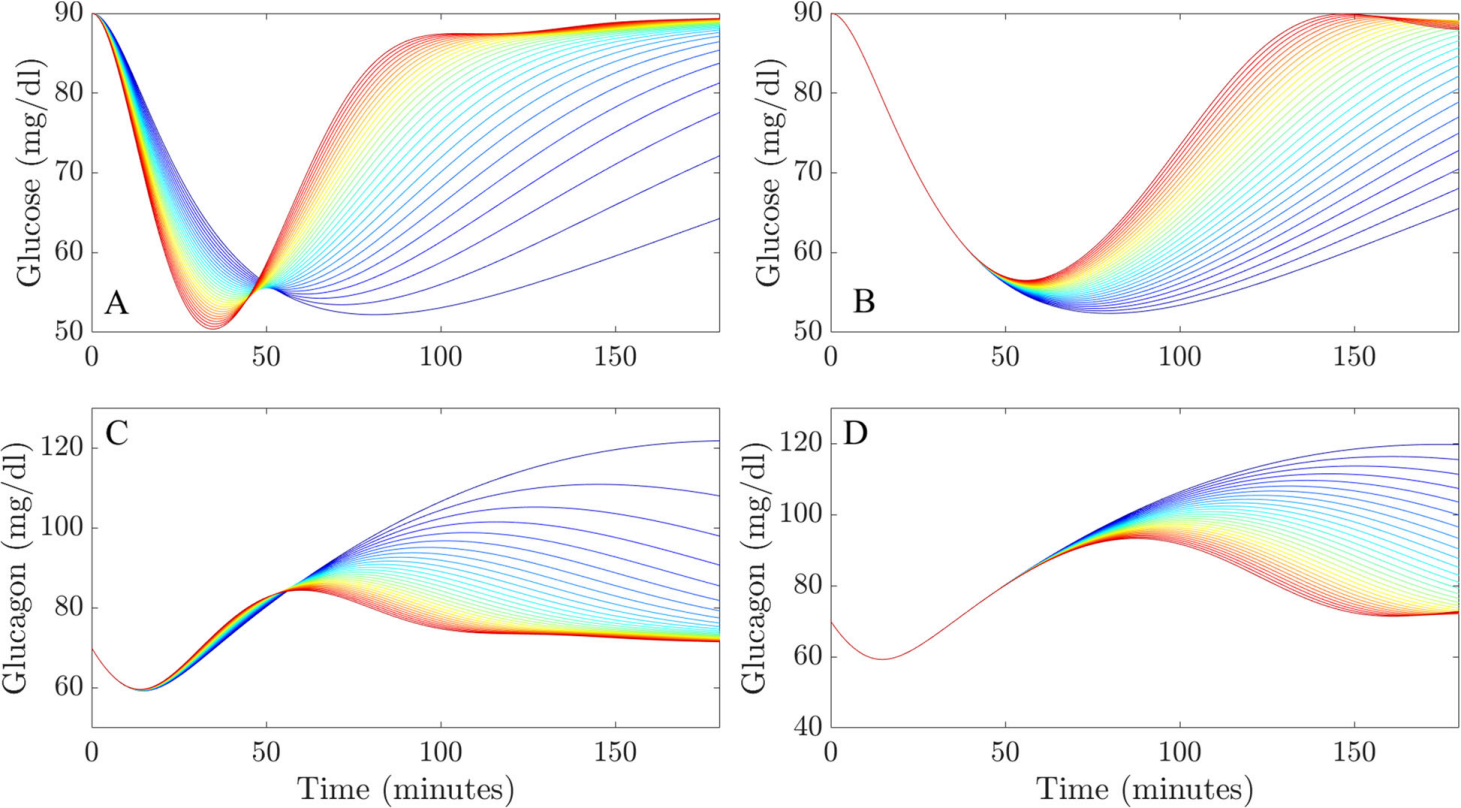
Statistical comparison of glucose effectiveness and insulin sensitivity. The evolution of blood glucose and plasma glucagon concentrations after an injection of one unit of insulin for patients with different degrees of glucagon sensitivity and glucagon effectiveness. A and C present the results from the LGMM, while B and D present the results from the NLGMM. The colour scheme indicates low glucagon effectiveness / sensitivity (dark blue) to high glucagon effectiveness / sensitivity. The value of *δ* varies between 0.0001 and 0.01 in increments of 0.005, whilst the value of *s*_*E*_ varies between 1×10^−5^ and 5×10^−4^ in increments of 2.45×10^−5^. All of the parameter values used for these simulations are located in Table 8 in Appendix D

Figure 7 shows the glucose and plasma glucagon concentration profiles of T1DM patients produced in this test. The LGMM predicts that a patient with a higher glucagon effectiveness will experience a rapid reduction in plasma glucose, followed by a quicker, full recovery to basal levels (Fig. 7a). Conversely, a patient who is glucagon ineffective, will fail to recover to pre-test glucose concentrations during the 180 min simulation. The NLGMM model predicts that there will be no difference between a glucagon sensitive or insensitive individual for the first 50 min of the test (Fig. 7b). However, glucagon effective patients will recover before 150 min, whereas patients who are glucagon ineffective will fail to recover to basal glucose concentrations. Outputs for both models are intuitive, with the predominant differences between the LGMM and NLGMM resting in the recovery time. The LGMM predicts glucagon sensitive individuals may recover rapidly, compared to the NLGMM, which predicts a much more delayed recovery time. Again, this behaviour is a function of how the LGMM and NLGMM each represent glucose metabolism, with the LGMM rate of change of glucose concentration being directly dependent on plasma glucagon, leading to an immediate fall in plasma glucose. The blood glucose concentrations presented here for a patient with a normal response to glucagon are qualitatively identical to those presented in [25] for patients with type 1 diabetes and normal response to glucagon which further validates the predictions produced from both models.

Figure 7 also presents the simulated plasma glucagon concentration profiles from the LGMM and NLGMM (7 C and 7 D), which are virtually identical in every case, indicating that an individual with very low glucagon effectiveness / sensitivity experiences a large increase in the concentration of plasma glucagon. The only difference between the LGMM and NLGMM, similar to the glucose concentration profiles, is the delayed recovery response-time of the NLGMM compared to the LGMM. It is further evident that type 1 diabetics with an impaired response to glucagon would be unable to raise their blood glucose levels and would require an infusion of glucose to recover from hypoglycemia. Type 1 diabetics with a normal response to glucagon however are able to recover from hypoglycemia without insulin infusion.

### Model considerations and applications

Both the LGMM and NLGMM fit well to the glucose, glucagon and insulin profiles from modified and glucose infusion IVGTT data. The ability of both models to replicate the data was compared using the AIC and BIC penalised-likelyhood criterion tests, which suggested that the LGMM is considered the superior model with respect to simulating an IVGTT, as well as for insulin infusion models. This finding was bolstered during the parameter re-estimation analysis, where the LGMM was statistically more accurate when predicting glucose effectiveness and insulin sensitivities for a “virtual patient cohort” given 0%, 5% and 10% noise. While the LGMM appears to best the NLGMM in terms of replicating IVGTT and insulin infusion data, simulations of blood glucose and glucagon concentrations in Figs. 6 and 7 present the merits of the linear and non-linear descriptions of glucagon metabolism. Simulations of both models allow prediction of how inter-individual variations in glucagon effectiveness and sensitivity can affect plasma glucose and glucagon concentrations. LGMM and NLGMM simulations of IVGTT and insulin infusion data stand strong compared to the Minimal Model ouputs in Figs. 2 and 3 for glucose and insulin outputs.

It is important to note that the driving force of this work was not to improve the accuracy of predicting glucose effectiveness (*s*_*G*_) and insulin sensitivity (*s*_*I*_) parameters with respect to the Minimal Model, but rather, to expand the mathematical metabolic portrait to include the role of glucagon, given the current surge of interest it has received in the field of diabetology.

## Conclusion

Presented here are two mathematical models of glucagon-glucose-insulin metabolism, used to simulate an IVGTT. The first, assumes a complex, non-linear glucose-glucagon-insulin relationship, while the second assumes that the rate of change of glucose concentration is proportional to the concentration of plasma glucagon. Both models accurately replicate insulin-modified and glucose infusion IVGTT data, while also being able to re-estimate the key physiological parameters, glucose effectiveness (*s*_*G*_) and insulin sensitivity (*s*_*I*_). Inclusion of glucagon dynamics allow estimation of two new parameters, glucagon sensitivity (*s*_*E*_) and glucagon effectiveness (*δ*), which describe the quantitative enhancement of glucose appearance due to an increase in plasma glucagon concentration. Perturbation of these parameters facilitates investigation of inter-individual variation of glucagon sensitivity and the resulting changes on plasma glucose and glucagon concentration. The LGMM and NLGMM allow the role of glucagon during an glucose tolerance testing and insulin infusion to be investigated, as well as providing a mathematical platform from which potential glucagon-based therapeutics may be explored.

## Appendix

## Appendix A: Model analysis

### Qualitative study of solutions

Due to the inherent non-linearity within both models, it is impossible to obtain analytical solutions of either system and numerical methods must be used instead to obtain approximate solutions. It is possible however to obtain qualitative information about the behaviour of solutions of both plasma insulin and plasma glucagon without being able to explicitly solve for *I*(*t*) and *E*(*t*). In what follows in this section, the qualitative behaviour of the NLGMM is discussed, as the equations modelling the concentration of plasma insulin and plasma glucagon are identical in both systems and a separate analysis of both systems here is unnecessary.

If one attempts to solve (3) in isolation from the rest of the NLGMM, the solution

20 $$\begin{array}{*{20}l} I(t)&=I_{b}+(I_{0}-I_{b})e^{-p_{4} t}\\ &+p_{5}e^{-p_{4} t}\int_{0}^{t}s(G(s)-G_{b})^{+}e^{p_{4} s}\text{ds}, \end{array} $$

is obtained for *t*≥0. This solution is of little practical use as *G*(*t*) is unknown but it can be immediately observed that when *G*(*t*)>*G*_*b*_,*I*(*t*)>*I*_*b*_ which is what should happen as insulin is released to counteract an increase in glucose. However, if *G*(*s*)<*G*_*b*_, the exact solution

21 $$\begin{array}{*{20}l} I(t)=I_{b}+(I_{0}-I_{b})e^{-p_{4} t} \end{array} $$

is obtained, which still satisfies *I*(*t*)≥*I*_*b*_. It may be deduced therefore that the equation modelling the concentration of plasma insulin in both models does not allow the concentration to drop below the basal level. This is also true of the Minimal Model.

The behaviour of plasma glucagon is more complex as it is assumed to depend on both glucose and insulin and the ODE is non-linear. However, when a patient is experiencing hyperglycemia and the concentration of plasma insulin is very high, (3) may be simplified into the following equation

22 $$\begin{array}{*{20}l} E'(t)=-p_{6}(E-E_{b})-p_{11}. \end{array} $$

This ODE possesses the exact solution

23 $$\begin{array}{*{20}l} E(t)=E_{b}-\frac{p_{11}}{p_{6}}\big(1-e^{-p_{6} t}\big), \end{array} $$

where the initial condition *E*(0)=*E*_*b*_ has been applied. As *t*→*∞*, this solution tends to the constant value

24 $$\begin{array}{*{20}l} E_{\infty}=E_{b}-\frac{p_{11}}{p_{6}}, \end{array} $$

which is the minimum possible concentration of plasma glucagon and a steady state solution. In order to ensure physiologically sensible solutions, we must have that

25 $$\begin{array}{*{20}l} \frac{p_{11}}{p_{6}}<E_{b}. \end{array} $$

The integral representations of the solutions of both interstitial insulin and glucagon activity are found to be

26 $$\begin{array}{*{20}l} X(t)&=e^{-p_{2} t}\int_{0}^{t}(I(s)-I_{b})e^{p_{2} s}\d s, \end{array} $$

27 $$\begin{array}{*{20}l} Y(t)&=e^{-p_{6} t}\int_{0}^{t}(E(s)-E_{b})^{+}e^{p_{6} s}\d s. \end{array} $$

Given that both integrands are non-negative for all possible values of *t*, it may be deduced that the concentrations of insulin and glucagon in tissue will always be non-negative. This is to be expected as it is clearly impossible to have negative concentrations of hormones in tissue, but it is reassuring that all model simulations are realistic in this sense.

### Investigating the existence of critical points

Determining the existence of steady-state solutions of both the NLGMM and LGMM is a useful exercise as such solutions allow characterisation of the long term behaviour of solutions obtained from both models.

As a system of differential equations can only possess one or more critical points if it is autonomous, it follows that the terms involving (*G*−*G*_*b*_)^+^ and (*G*_*b*_−*G*)^+^ must vanish simultaneously to suppress the explicit appearance of the time variable. From this information, one can deduce that the only possible critical point of the NLGMM is

28 $$\begin{array}{*{20}l} (G^{\star},X^{\star},I^{\star},E^{\star},Y^{\star})=(G_{b},0,I_{b},E_{b},0). \end{array} $$

It similarly follows that the only critical point of the LGMM is

29 $$\begin{array}{*{20}l} (G^{\star},X^{\star},I^{\star},E^{\star})=(G_{b},0,I_{b},E_{b}). \end{array} $$

In both cases, the critical point corresponds to the physical situation of the patient not being administered glucose and thus their body remaining in the fasting state.

### Classifying the long term behaviour of model solutions

Having found the critical points of both systems, it is now of interest to classify their nature and determine how model solutions behave in the limit *t*→*∞*. The stability of these critical points may be determined by evaluating the Jacobian matrices of both systems of equations at the critical point (see [27] for example). In the case of the NLGMM, the requisite matrix is

30 $$\begin{array}{*{20}l} J^{\star}=\left(\begin{array}{ccccc} -p_{1} & -G_{b} & 0 & 0 & G_{b}\\ 0 & -p_{2} & p_{3} & 0 & 0\\ 0 & 0 & -p_{4} & 0 & 0\\ 0 & 0 & -\alpha p_{11} & -p_{6} & 0\\ 0 & 0 & 0 & p_{9} & -p_{8}\\ \end{array}\right) \end{array} $$

Attempting to solve for the eigenvalues of this matrix reveal that they satisfy the equation

31 $$\begin{array}{*{20}l} \left(p_{1}+\lambda\right)\left(p_{2}+\lambda\right)\left(p_{4}+\lambda\right)\left(p_{6}+\lambda\right)\left(p_{8}+\lambda\right)=0, \end{array} $$

and hence

32 $$\begin{array}{*{20}l} \lambda=-p_{1},-p_{2},-p_{4},-p_{6},-p_{8}. \end{array} $$

As all model parameters appearing in the NLGMM are positive, these eigenvalues are negative and hence the critical point is stable. This means that all solutions produced from this model will eventually return to the pre-test fasting levels obtained for a patient.

In the case of the LGMM, the Jacobian matrix evaluated at the critical point is

33 $$\begin{array}{*{20}l} J^{\star}=\left(\begin{array}{cccc} -p_{1} & -G_{b} & 0 & \delta\\ 0 & -p_{2} & p_{3} & 0\\ 0 & 0 & -p_{4} & 0\\ 0 & 0 & -\alpha p_{11} & -p_{6}\\ \end{array}\right), \end{array} $$

and the eigenvalues of this matrix are

34 $$\begin{array}{*{20}l} \lambda=-p_{1},-p_{2},-p_{4},-p_{6}. \end{array} $$

As all obtained eigenvalues are again negative here, this critical point is also stable and all model solutions will eventually return to a patient’s pre-test fasting levels.

## Appendix B: Modelling an IM-IVGTT and GC-IM-IVGTT

In a standard IVGTT, glucose is administered intraveously only at the beginning of the test and there is no infusion of insulin. In this situation, the Minimal Model and both the LGMM and NLGMM assume that the initial concentrations of plasma glucose and plasma insulin are very high at the beginning of the test. These assumptions have the advantage of simplicity and have proven to produce accurate predictions in numerous investigations.

The infusion of insulin that is given after 20 min in both the IM-IVGTT and GC-IM-IVGTT needs to be directly accounted for within any approximating model to ensure that the predicted glucose response is accurate. This requires the use of a suitable function *I*_*inf*_(*t*) to model this dose. Based on the details provided about the average patient BMI and dosage given in [17], the insulin infusion is modelled using the following function:

35 $$\begin{array}{*{20}l} I_{inf}(t)=3920\hspace{0.1cm} e^{-8|t-20|}. \end{array} $$

This function has been chosen as it represents a dose administered over a maximum of 60 s. The value 3920 was determined by LSQNONLIN when this function was fitted to the dataset for plasma insulin given in the IM-IVGTT.

In the GC-IM-IVGTT, Thomaseth et al. do not give any indication as to how much glucose is infused to prevent hypoglycaemia, rather. Instead, the authors indicate that the additional glucose infusion is adjusted manually to prevent hypoglycaemia. In the absence of more specific information, it is assumed that when glucose concentration reaches 100 mg /dl, it ceases to vary, and hence (1) is replaced by the alternate equation:

$$\begin{array}{*{20}l} G'(t)=0. \end{array} $$

Whilst this is not a true representation of how the glucose concentration actually behaves, it does make it possible to examine whether the simulated behaviour of glucagon is qualitatively correct or not in this case.

## Appendix C: Modelling an insulin infusion test

In an insulin infusion test, insulin is administered intravenously at a constant rate for a substantial period of time. Within this work, the insulin infusion term within Eqs. (3) and (10) is chosen to be

$$\begin{array}{*{20}l} I_{\text{inf}}(t)= \left\{\begin{array}{cc} I_{d},\qquad 0\leq t\leq65,\\ 0, \qquad t>65, \end{array}\right. \end{array} $$

where *I*_*d*_ is the provided dosage of insulin with corresponding units *μU*/ml min ^−2^. This value may be computed using the information regarding dosage provided by [25].

The initial conditions for both the LGMM and NLGMM are also much simpler in an insulin infusion test as the initial concentrations of blood glucose and plasma insulin are assumed to be at their basal level. The initial conditions for the NLGMM in this case are:

36 $$\begin{array}{*{20}l} G(0)&=G_{b}, \qquad X(0)=0, \qquad I(0)=I_{b},\\ & \qquad E(0)=E_{b}, \qquad Y(0)=0, \end{array} $$

and the initial conditions for the LGMM are

37 $$\begin{array}{*{20}l} G(0)\,=\,G_{b}, \qquad X(0)\,=\,0, \qquad I(0)\,=\,I_{b}, \qquad E(0)\,=\,E_{b}. \end{array} $$

As a result, only 10 unknown parameters appear in the LGMM and 11 unknown parameters appear in the NLGMM in this test.

## Appendix D: Simulation parameters

The following Table contains the parameters used to investigating the response of glucagon during an IVGTT.

**Table 6:** The range of parameter values used to create a virtual cohort of patient data

Parameter	Median value	Smallest value	Largest value
*p*_1_	0.01	0.001	0.1
*p*_2_	0.05	0.01	0.9
*p*_3_	1×10^−5^	1×10^−6^	1×10^−4^
*p*_4_	0.225	0.05	0.4
*p*_5_	0.005	0.001	0.009
*p*_6_	0.055	0.01	0.1
*p*_7_	1×10^−4^	1×10^−5^	1×10^−3^
*p*_8_	0.26	0.1	0.52
*p*_9_	5×10^−3^	1×10^−4^	9×10^−3^
*p*_11_	0.125	0.5	2
*δ*	0.01	0.001	0.1
*G*_*b*_	85	70	100
*I*_*b*_	12	7	17
*E*_*b*_	65	50	85

The corresponding units for each quantity are described in Table 1

**Table 7:** Parameter values used in the example investigating glucagon suppression caused by insulin

Parameter	Variable (Yes/no)	Value
*G*_*b*_	No	95
*I*_*b*_	No	90
*E*_*b*_	No	70
*p*_1_	No	0.01
*p*_2_	No	0.015
*p*_3_	No	6×10^−4^
*p*_4_	No	0.1
*p*_5_	No	0.0045
*p*_6_	No	0.08
*p*_7_	No	9×10^−4^
*p*_8_	No	0.13
*p*_9_	No	6.5×10^−5^
*p*_11_	Yes	[0.01,2]
*δ*	No	0.1
*G*_0_	No	270
*I*_0_	No	325

The corresponding units for each quantity are described in Table 1

**Table 8:** Parameter values used in the example investigating the response of glucagon and glucose due to an infusion of insulin

Parameter	Variable (Yes/no)	Value
*G*_*b*_	No	95
*I*_*b*_	No	0
*E*_*b*_	No	70
*p*_1_	No	0.01
*p*_2_	No	0.015
*p*_3_	No	6×10^−4^
*p*_4_	No	0.1
*p*_5_	No	0.0045
*p*_6_	No	0.08
*p*_7_	No	9×10^−4^
*p*_8_	No	0.13
*s*_*E*_	Yes	[0.00001,0.0005]
*p*_11_	No	1.5
*δ*	Yes	[0.0001,0.1]

The corresponding units for each quantity are described in Table 1

## Data Availability

All model parameters and kinetic information are presented in the Appendix.
